# Macroscopic quantum effects in the brain: new insights into the fundamental principle underlying conscious processes

**DOI:** 10.3389/fnhum.2025.1676585

**Published:** 2025-12-03

**Authors:** Joachim Keppler

**Affiliations:** Department of Consciousness Research, DIWISS Research Institute, Roth, Germany

**Keywords:** conscious processes, fundamental principle, self-organized criticality, phase transitions, quantum electrodynamics, macroscopic quantum effects, coherence domains, zero-point field

## Abstract

Empirical findings indicate that conscious states are inextricably linked to long-range synchronized activity patterns that result from phase transitions and exhibit the key features of self-organized criticality. This article builds a bridge between these neurophysiological characteristics of consciousness and the framework of quantum electrodynamics (QED), which provides the appropriate methodological resources for explaining the origin of phase transitions and critical dynamics. An essential ingredient of QED is a fluctuating ocean of energy, the ubiquitous electromagnetic zero-point field (ZPF), consisting of a spectrum of normal modes. It can be deduced from QED-based model calculations that the resonant interaction of the ZPF with the glutamate pool of cortical microcolumns is an important prerequisite for the initiation of phase transitions, giving rise to macroscopic quantum effects that play a crucial role in modulating the activity of ion channels and regulating the neuronal firing rate. The firing rate of pyramidal neurons and inhibitory interneurons determines the excitatory-inhibitory balance, which has been identified as the essential control parameter for establishing and maintaining the critical regime. Thus, taking all available pieces of evidence into account, profound new insights take shape, namely, that self-organized criticality arises from a bottom-up orchestration process involving the ZPF and that the fundamental principle behind the formation of conscious states is the resonant coupling of the brain to the ZPF. This coupling causes an amplification of the dynamically relevant ZPF modes, suggesting that the ZPF holds the key to the understanding of consciousness and that the necessary condition for the formation of a conscious state is the selective excitation of ZPF modes. These insights pave the way for novel experimental paradigms designed to systematically manipulate conditions in the brain, thereby collecting new data that can be used to empirically substantiate the significance of resonant brain-ZPF interaction for the formation of conscious states.

## 1 Introduction

A central element of human existence is our conscious experience, which is distinguished by an enormous range of differentiated conscious states. Conscious processes include both stimulus-induced conscious perception and stimulus-independent mental processes, such as thinking and memory retrieval. Over the past few decades, a wealth of data has been accumulated on these brain processes, providing us with a substantial empirical body of empirical evidence on the neurophysiological characteristics of consciousness. The challenge now is to exploit the available evidence to infer the core principle that is effective in conscious processes but absent in unconscious processes. Proceeding from the inferred operating principle, insights can be gained into the necessary conditions that must be fulfilled in the brain for conscious states to arise. The specification of this principle and the formulation of these conditions are essential prerequisites for the development of a solid theory of consciousness that we expect to possess explanatory power and unveil causal connections. In turn, knowledge of causal dependencies is important for exploring new research avenues and setting up innovative experiments that are designed to produce additional data by systematically modifying the local conditions in the brain, with the goal of underpinning the inferred operating principle behind conscious processes. Ideally, the revealed principle turns out to be universal, so that conclusions can be drawn about the potential presence of consciousness in other species.

This article aims to shed light on the fundamental principle underlying conscious processes and to provide insight into the necessary conditions that must be satisfied in the brain to form conscious states. This is achieved by putting together all the essential pieces of the puzzle, including brain architectural and neurophysiological findings as well as quantitative model calculations. The result of this novel synthesis is a precise characterization of the conditions that differentiate conscious processes from unconscious brain activity. As we will see, the overall picture suggests that macroscopic quantum effects play a significant role in the formation of those neural activity patterns that are associated with conscious states, while these quantum effects are not present during periods of unconsciousness.

Our focus here is entirely on the *neurophysiology of consciousness*, while the metaphysics of consciousness is not the subject of the present work, nor is the formulation of a full-fledged theory of consciousness that incorporates the phenomenological aspects of awareness. Concerning the metaphysical implications of the presented insights and the conceptual considerations that pave the way for the development of a self-consistent theory of consciousness, the reader is referred to previous works in which these matters are discussed in detail (Keppler, 2016, 2018, 2021, 2024; Shani and Keppler, 2018; Keppler and Shani, 2020).

The article is organized in such a way that we first address the neurophysiological characteristics of consciousness. We will then take an in-depth look at the operating principle of cortical microcolumns, which constitute the basic functional units of the cortex. Drawing on this deep understanding, we proceed to throw light on the fundamental principle underlying conscious processes and to describe experimental strategies by which the principle can be further empirically corroborated. We will conclude with a brief discussion and an outlook.

## 2 Neurophysiological characteristics of consciousness

**Figure 1:**
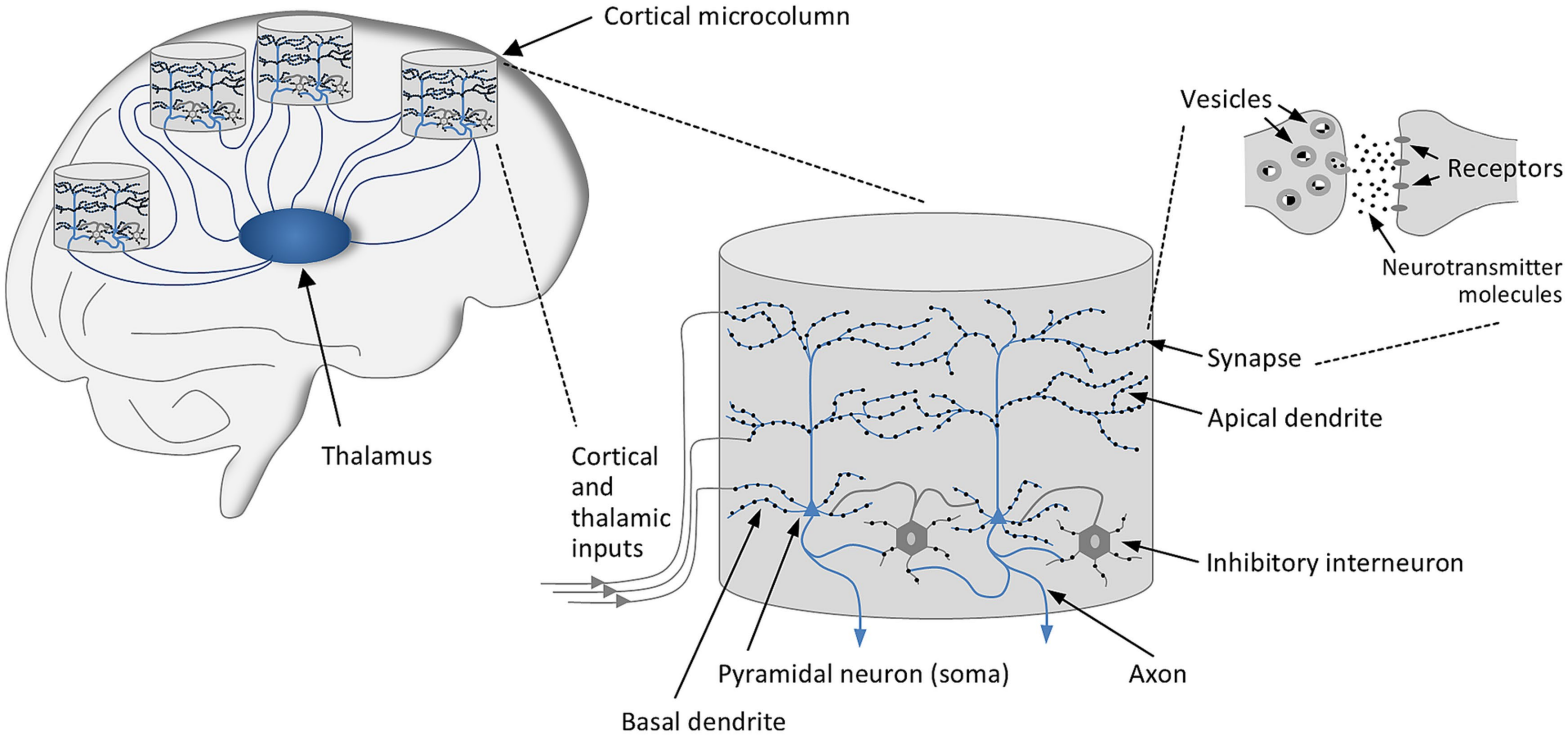
Brain architecture and organization of the cerebral cortex. The cortex is organized in modules, with the cortical microcolumn constituting the basic functional unit. Each microcolumn consist of roughly 100 neurons, reaches a diameter of the order of 30 μm, and receives numerous inputs from other cortical areas and the thalamus. Corticocortical and thalamocortical fibers project to tens of thousands of excitatory, mostly glutamatergic, synapses that are densely distributed across the basal and apical dendrites of the pyramidal neurons, which represent about 80% of all neurons. The remaining neurons are mainly inhibitory interneurons, which regulate the activity of pyramidal neurons via GABAergic synapses.

Let us first turn to the architecture of the brain and the organization of the cerebral cortex (see Figure 1). Experimental findings suggest that the cortex, which is formed of layers, can be viewed as a modular system whose basic functional unit is the minicolumn, also termed *microcolumn* (Kohn et al., 1997; Mountcastle, 1997; Buxhoeveden and Casanova, 2002; Hosoya, 2019). Even though the microcolumns, which consist of roughly 100 neurons and reach a diameter of approximately 30 μm, differ in their details, they all share a uniform design (Jones, 2000; Buxhoeveden and Casanova, 2002). The microcolumns are highly interconnected, just as there is also a high degree of connectivity between the microcolumns and subcortical structures, especially the thalamus (Mountcastle, 1997). Each microcolumn receives numerous inputs from other cortical areas and thalamic modules, with corticocortical and thalamocortical fibers transmitting signals to the tens of thousands of excitatory, mostly glutamatergic, synapses that are densely distributed across the basal and apical dendrites of the pyramidal neurons, which play a major role in microcolumns and represent about 80% of all neurons (Spruston, 2008). The remaining neurons are mainly inhibitory interneurons, which regulate the activity of pyramidal neurons via GABAergic synapses and, in concert with the pyramidal cells, generate oscillatory network activity (Buzsáki and Wang, 2012; Schmidt-Wilcke et al., 2018).

Empirical evidence indicates that conscious states are closely linked to *long-range synchronized* brain activity in the beta and gamma frequency bands (Rodriguez et al., 1999; Engel and Singer, 2001; Melloni et al., 2007; Gaillard et al., 2009; Uhlhaas et al., 2009; Steinmann et al., 2014). During conscious perception, the formation of synchronized activity patterns follows the theta rhythm (Freeman, 2004; Doesburg et al., 2009), while during stimulus-independent conscious processes, the pattern formation cycle corresponds to the alpha rhythm (Freeman, 2004; Knyazev et al., 2011). A more in-depth analysis of the data suggests that synchronized activity patterns result from *phase transitions* and represent the *collective behavior* of large numbers of neurons (Kelso et al., 1992; Freeman, 2004, 2007). In all frequency bands, pattern formation exhibits long-range correlations and robust features of *criticality* (Linkenkaer-Hansen et al., 2001; Freeman, 2004, 2007; Kitzbichler et al., 2009; Chialvo, 2010; Tagliazucchi et al., 2012). In line with these findings, recent studies demonstrate that consciousness is supported by activity patterns displaying the characteristics of critical dynamics, while under anesthetic conditions that induce unconsciousness, brain dynamics deviate from criticality, implying that criticality is a necessary requirement for the emergence of conscious states (Tagliazucchi et al., 2016; Toker et al., 2022; Maschke et al., 2024).

Viewed from a different perspective, the nested theta and beta/gamma oscillations that occur in the context of conscious perception bear the hallmarks of neuronal avalanches (Gireesh and Plenz, 2008). These avalanches, which represent the propagation of locally synchronized action potentials and give rise to large-scale activity patterns extending across a great number of cortical microcolumns, feature long-range correlations, indicative of a system operating in the critical regime (Beggs and Plenz, 2003; Plenz and Thiagarajan, 2007; Lombardi et al., 2014). The corresponding operating principle is known as *self-organized criticality* (SOC), which refers to a system’s ability to adjust a control parameter that keeps the system in the vicinity of a critical point of a phase transition (Plenz et al., 2021). In the brain, the key control parameter appears to be the excitatory-inhibitory (E-I) balance, involving the neurotransmitters glutamate and gamma-aminobutyric acid (GABA) as well as the neuromodulators dopamine, acetylcholine, and serotonin (Plenz et al., 2021). The neuromodulators affect avalanche dynamics, with dopamine having been shown to facilitate avalanche formation by promoting the depolarization of interneurons and regulating the interaction of pyramidal neurons and interneurons (Stewart and Plenz, 2006; Plenz et al., 2021). With predefined neuromodulatory settings (as they exist in a certain brain state, such as wakefulness), it turns out that the emergence of neuronal avalanches, which correspond to coherent beta/gamma activity and are characterized by critical dynamics, requires the coordinated interplay of excitatory NMDA-type glutamate receptor-mediated synaptic transmission and inhibitory GABA_A_ receptor-mediated synaptic transmission (Mazzoni et al., 2007; Gireesh and Plenz, 2008).

In summary, the body of empirical evidence provides some important pointers to the principles underlying conscious processes and the necessary conditions for the formation of conscious states. The evidence suggests that conscious states are associated with coherent activity patterns displaying collective behavior and involving large numbers of cortical microcolumns, that each activity pattern originates from a phase transition, and that the induction of phase transitions and the control of avalanches are achieved by a well-orchestrated concert of neurotransmitter releases and neuronal discharges. It is this orchestration that is at the root of critical dynamics and needs to be explored in greater detail. In this sense, the approach taken here is in accordance with considerations that assume criticality and self-organization to be essential for understanding the neurophysiological characteristics of conscious processes (Fingelkurts et al., 2013) and see SOC as a promising candidate for a unified theoretical framework for consciousness (Walter and Hinterberger, 2022).

To fully grasp the significance of these clues and unveil the fundamental principle behind conscious processes, we must advance to a deeper level of explanation. This leads us into the realm of quantum field theory, equipping us with the appropriate methodological resources for explaining phase transitions and understanding collective behavior in many-body systems (Del Giudice et al., 1985, 2005; Freeman and Vitiello, 2006, 2008). In concrete terms, we will utilize the framework of *quantum electrodynamics* (QED), the fundamental theory of electromagnetism, to gain insight into the functional principle of cortical microcolumns.

## 3 Functional principle of cortical microcolumns

Returning to the central role of neurotransmitters in the initiation of phase transitions, we focus on glutamate, by far the most abundant neurotransmitter in the brain, whose concentration in neural tissue is not only significantly higher than that of GABA and the neuromodulators but even exceeds the levels of all other molecular components except water (Featherstone, 2010). While the peak concentration of glutamate is localized in synaptic vesicles, regulatory processes along the glutamate-glutamine cycle, taking place in astrocytes, maintain an average microcolumnar glutamate concentration that varies from brain region to brain region and represents the overall intracellular glutamate pool of a microcolumn (Erecińska and Silver, 1990; Featherstone, 2010). Thus, from a physical point of view, a microcolumn can be regarded as a network of pyramidal neurons and interneurons integrated into a *glutamate pool* that, to be precise, forms a glutamate-water matrix (Keppler, 2024). In this model, we disregard the layered structure of the cortex; however, this has no impact on disentangling the functional principle of an individual microcolumn and will not prevent us from understanding how neuronal avalanches are triggered and controlled (Keppler, 2023, 2024).

Starting from this slightly simplified model of a cortical microcolumn, we bring QED into play, according to which the vacuum, rather than being an empty space, is pervaded by a fluctuating ocean of energy that plays a central part in the worldview of modern physics. One constituent of this fluctuating ocean is the electromagnetic *zero-point field* (ZPF), consisting of a spectrum of normal modes, each of which is characterized by a specific frequency. Approaches that delve into the conceptual foundations of quantum physics suggest that the origin of all quantum phenomena lies in the ZPF, and that this omnipresent energy field is an *essential ingredient of nature’s blueprint* (De la Peña and Cetto, 1994, 1995; De la Peña et al., 2015; Cetto and de la Peña, 2022).

The formalism of QED has proven to be highly effective in describing the interaction of a many-body system with the ZPF (Preparata, 1995; Del Giudice et al., 2005; Del Giudice and Vitiello, 2006). Applying this formalism, a mathematical description of a microcolumn is obtained that includes the *coupling of the ZPF to the glutamate pool*. Such a description results in equations expressing the dynamical evolution of the coupled system (Keppler, 2023, 2024), indicating that the functional role of the glutamate pool of a microcolumn goes far beyond glutamate receptor-mediated synaptic signal transmission. In the following, the main findings of the model calculations are summarized and discussed.

**Figure 2:**
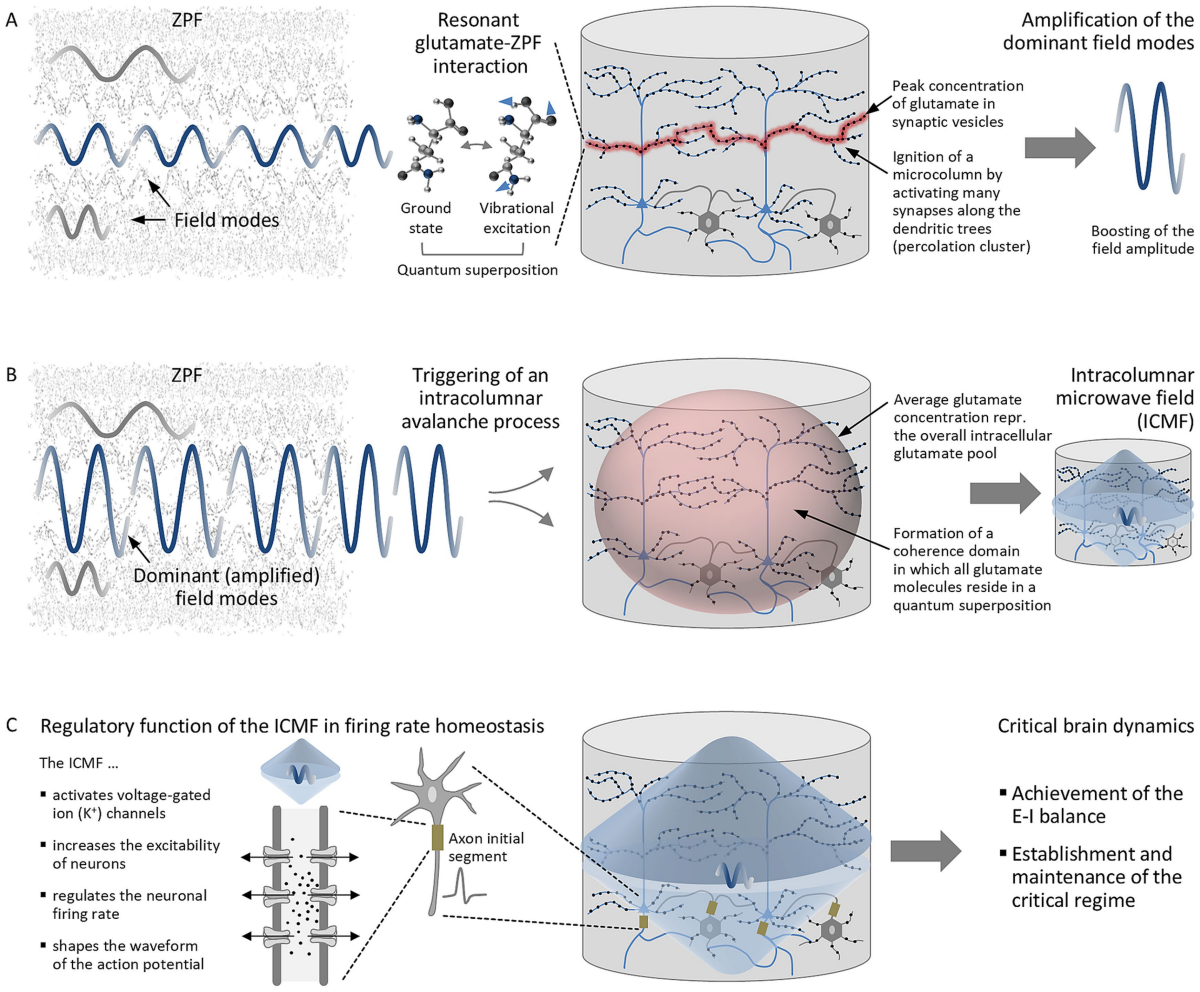
Functional principle of a cortical microcolumn according to a QED-based model. **(A)** A phase transition is initiated by resonant glutamate-ZPF interaction in synaptic vesicles, in which the glutamate concentration reaches its peak value. The resonant interaction leads to quantum superposition on the part of the glutamate molecules and, on the part of the ZPF, to the amplification of the dominant field modes. **(B)** The dominant, considerably amplified ZPF modes kick off an intracolumnar avalanche process that drives a microcolumn’s entire glutamate pool to a macroscopic quantum state, resulting in the formation of a coherence domain and the generation of an intracolumnar microwave field (ICMF). **(C)** The ICMF modulates the activity of voltage-gated ion channels and plays an important role in the homeostatic regulation of the neuronal firing rate, which has been identified as the prerequisite for establishing the E-I balance and maintaining the critical regime.

According to the evolution equations, the dynamical properties of the system are determined by the coupling strength between the two players, where the coupling strength is directly related to the glutamate concentration. The calculations reveal that upon exceeding a critical glutamate concentration, the glutamate pool undergoes a *phase transition* prompted by a *resonance phenomenon* that favors one of the excited states (vibrational excitations) of the glutamate molecules, subsequently termed preferred vibrational excitation, whereupon those ZPF modes dominate the evolution whose energy corresponds to the energy difference between the ground state and the preferred excited state of the molecules, subsequently referred to as dominant ZPF modes. These findings, which are supported by a comprehensive mathematical analysis (Keppler, 2023, 2024), suggest that the functional principle of a cortical microcolumn is based on a three-stage process (see Figure 2).

In the first stage, displayed in Figure 2A, a *phase transition is initiated by resonant glutamate-ZPF interaction*. Such a phase transition is triggered in synaptic vesicles, in which the glutamate concentration reaches its peak value. The computations indicate that this peak value lies exactly at the critical threshold required to set off the resonance phenomenon (Keppler, 2023). The resonant coupling of the ZPF to vesicular glutamate has two consequences, namely, on the one hand, the emergence of a quantum state in which the glutamate molecules exist in a superposition of the ground state and the preferred vibrational excitation, and, on the other hand, the *amplification of the dominant ZPF modes*, reflected in a considerable enhancement of the field amplitude. The calculations yield a value of 7.8 THz for the frequency of the dominant ZPF modes (Keppler, 2023).

To induce a phase transition that extends across the full width of a microcolumn, tantamount to the ignition of a microcolumn, many synapses along the dendritic trees need to be activated, causing a large number of synaptic vesicles to release highly concentrated glutamate clouds, which then combine into a percolation cluster (Keppler, 2023, 2024). For one thing, this ignition criterion provides a plausible explanation for the special architecture of pyramidal neurons, featuring extensive apical dendrites that are studded with excitatory synapses (Spruston, 2008), and for another thing, the importance of the thalamus for waking consciousness becomes apparent from the ignition criterion, as thalamocortical pathways relay sensory stimuli to cortical microcolumns, innervating tens of thousands of glutamatergic synapses located on the apical dendrites of cortical pyramidal neurons (Modolo et al., 2020).

After the ignition of a microcolumn, the dominant, considerably amplified ZPF modes kick off the second stage, depicted in Figure 2B. The distinctive feature of this stage is an *intracolumnar avalanche process* that drives a microcolumn’s entire glutamate pool to a macroscopic quantum state. Crucially, the transition to this stationary state results in the *formation of a coherence domain* in which all glutamate molecules reside in a quantum superposition. The specific properties of a coherence domain are dictated by the frequency of the dominant ZPF modes and the average glutamate concentration of a microcolumn.

These properties include, firstly, the diameter of a coherence domain, which, according to the calculations, amounts to approximately 30 μm (Keppler, 2023). This value corresponds excellently with the experimentally verified width of a microcolumn (Jones, 2000).

Secondly, it follows from the stationary state equation of a coherence domain that the macroscopic quantum state of the glutamate pool, in which roughly 10^11^ molecules are involved, is energetically lowered, giving rise to a substantial energy gap that protects a coherence domain from thermal influences and inhibits rapid decoherence (Keppler, 2023). The significance of such an energy gap for maintaining a quantum superposition is corroborated by other studies (Del Giudice et al., 2005; Mewes et al., 2005; Del Giudice and Vitiello, 2006; Rey et al., 2008; Bouganne et al., 2020). The protection of a coherence domain is due to the fact that the molecules of the glutamate-water matrix populate a collective vibrational state that is maintained by the strong coupling to the ZPF. Such a state is very robust against external perturbations, as the molecules prone to thermal collisions are only situated in the marginal zone of a domain (Del Giudice et al., 2005). Therefore, only a small fraction of the molecules is exposed to disruptive influences, and the calculations show that the cumulative disruptive energy transferred into a coherence domain via these molecules at body temperature is not sufficient to overcome the energy gap and break the collective state (Keppler, 2023). It should be emphasized that the presence of water supports the stabilization of the macroscopic quantum state, since water itself forms coherent structures (Arani et al., 1995; Bono et al., 2012; Del Giudice et al., 2013). The energy gaps of these structures pile up and make an additional contribution to the total energy gap of a coherence domain, suggesting that *macroscopic quantum coherence is feasible under the conditions found in cortical microcolumns*. These results highlight that a QED-based approach, which takes into account the importance of the ZPF for the emergence of macroscopic quantum states, leads to completely different conclusions regarding the plausibility of quantum coherence in biological organisms than purely quantum mechanical considerations that do not include the ZPF (Tegmark, 2000; Koch and Hepp, 2006).

Finally, the numerical analysis reveals that the formation of a coherence domain implicates a frequency shift of the dominant, considerably amplified ZPF modes from 7.8 THz to the microwave range near 30 GHz, implying that the second stage of the three-stage process culminates in the generation of an *intracolumnar microwave field* (ICMF), which is an endogenous, phase-transition-induced radiation field (Keppler, 2023).

The ICMF controls the third stage, depicted in Figure 2C. Both theoretical calculations and experimental studies demonstrate that microwaves strongly influence the flow of ions through voltage-gated ion channels and, in particular, modulate the flow rate in voltage-gated K^+^ channels (Pickard and Rosenbaum, 1978; Li et al., 2014). Due to the activation of K^+^ channels and the resulting increase in neuronal excitability, microwaves cause significant changes in the firing rate of neurons (Pikov et al., 2010). The finding that the firing rate of neurons is regulated by modulating the activity of ion channels is well confirmed (Marder and Goaillard, 2006). It is precisely this homeostatic regulation of the neuronal firing rate that has been identified as the mechanism underlying the E-I balance (Páscoa dos Santos and Verschure, 2025), which acts as essential control parameter behind SOC (Plenz et al., 2021), in line with a study indicating that firing rate homeostasis is the basis for critical dynamics (Ma et al., 2019). More specifically, empirical evidence suggests that firing rate homeostasis of fast-spiking parvalbumin-expressing (PV+) inhibitory interneurons, which constitute the largest subclass of cortical interneurons and are crucial for gamma oscillations (Bartos et al., 2007), plays an important part in the establishment and active maintenance of the critical regime (Ma et al., 2019). The spiking characteristics of PV + interneurons can be attributed to fast-acting K^+^ channels, the activation of which gives rise to a significantly compressed waveform of the evoked action potential (Milicevic et al., 2023). Revealingly, microwaves are able to activate K^+^ channels and produce the compressed waveform (Pikov et al., 2010), which is indicative of the *leading role of the ICMF in the regulation of the neuronal firing rate and the emergence of critical dynamics*.

Further clues to the regulatory function of the ICMF arise from the homeostatic mechanisms that stabilize the excitability and firing rate of neurons during development. On the one hand, empirical studies show that the homeostatic regulation of the firing threshold is accomplished by intrinsic plasticity, which manifests itself in modulating the density of voltage-gated ion channels in the axon initial segment (Marder and Goaillard, 2006; Wu et al., 2020; Wen and Turrigiano, 2024). On the other hand, calculations demonstrate that microwaves acting on the cell membrane, due to resonances in the range of tens of GHz, cause redistribution and density modulation of the ion channels in the axon initial segment, resulting in a significant alteration of the excitation threshold (Shneider and Pekker, 2014). Both findings together suggest that the ICMF is of central importance for the self-regulation of the neuronal firing rate, which is compatible with the cytoelectric coupling hypothesis, stating that electric fields guide neural activity (Pinotsis et al., 2023).

In summary, the collected clues add up to a consistent picture, supporting the notion that the functioning of cortical microcolumns is dominated by macroscopic quantum effects. More precisely, the evidence indicates that the operating principle of microcolumns is based on a *bottom-up orchestration process driven by the ZPF* (Keppler, 2023, 2024). The process starts with the initiation of a phase transition via resonant glutamate-ZPF interaction. Such a phase transition culminates in an intracolumnar avalanche process that gives rise to the formation of a coherence domain, reflected in a macroscopic quantum state of the glutamate pool, and results in the generation of an endogenous microwave field, the ICMF. The ICMF serves as a unique recognition signal and assumes a central control function within a microcolumn, namely, the modulation of the activity of ion channels, which influences the firing threshold of the neurons and regulates the neuronal firing rate. The homeostatic regulation of the firing rate has been identified as the prerequisite for keeping the E-I balance and maintaining the critical regime.

## 4 Fundamental principle underlying conscious processes

**Figure 3:**
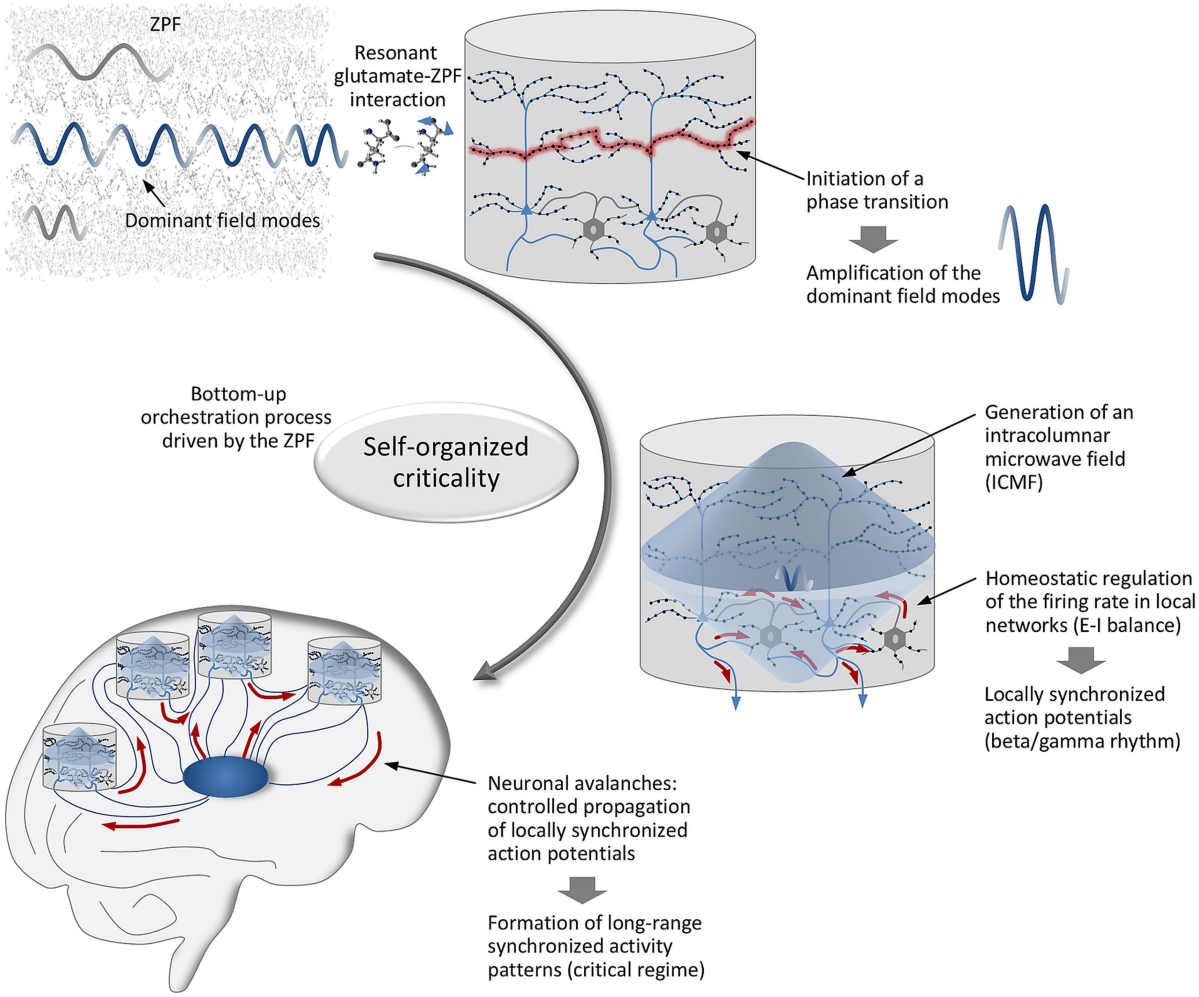
Self-organized criticality arising from a bottom-up orchestration process driven by the ZPF. Resonant glutamate-ZPF interaction triggers a phase transition in those microcolumns where the ignition criterion is satisfied. The phase transition is accompanied by an amplification of the dominant ZPF modes. Upon reaching a stationary state, the amplified modes build up an intracolumnar microwave field (ICMF) that plays a leading role in the homeostatic regulation of the neuronal firing rate and the establishment of the E-I balance, resulting in locally synchronized action potentials following the beta/gamma rhythm. The outcome is the controlled propagation of synchronized action potentials (neuronal avalanches) and the formation of synchronized activity patterns that bear the hallmarks of critical dynamics.

The intracolumnar, homeostatic regulation of the firing rate in the local network of pyramidal neurons and PV + inhibitory interneurons produces synchronized action potentials following the beta/gamma rhythm. To be exact, the discharge of pyramidal cells triggers the spiking of interneurons, which in turn inhibit the pyramidal cells and, by establishing a brief window of excitability, give rise to synchronized rhythmic activity (Fries et al., 2007; Salkoff et al., 2015). Building on this mechanism, a periodic, precisely controlled propagation of locally synchronized action potentials to other cortical areas and thalamic modules takes place. This propagation corresponds to the observed neuronal avalanches, leading to synchronized activity patterns that extend over large areas of the brain and bear the hallmarks of critical dynamics (Beggs and Plenz, 2003; Plenz and Thiagarajan, 2007; Gireesh and Plenz, 2008; Lombardi et al., 2014). The inferred causal chain behind the emergence of synchronized activity patterns is illustrated in Figure 3. An integral element of this chain is the ZPF, supporting the view that *SOC rests on the coupling of the brain to the ZPF*.

With these findings in mind, let us now close the circle and revisit the neurophysiological signatures of conscious and unconscious processes. Empirical evidence suggests that it is specifically the occurrence of long-range synchronized activity patterns in the beta and gamma frequency bands that is indicative of conscious states (Rodriguez et al., 1999; Engel and Singer, 2001; Melloni et al., 2007; Gaillard et al., 2009; Uhlhaas et al., 2009; Steinmann et al., 2014). Furthermore, it turns out that the synchronized activity patterns involved in consciousness exhibit the characteristics of critical dynamics, while a departure from the critical regime is associated with periods of unconsciousness (Alonso et al., 2014; Tagliazucchi et al., 2016; Toker et al., 2022; Maschke et al., 2024). Drawing on the causal connections presented above, these signatures indicate that the ZPF is instrumental in orchestrating those activity patterns that are related to consciousness, while in unconscious processes the coordinating role of the ZPF is lacking or impaired, supporting the conclusion that the formation of conscious states depends on resonant brain-ZPF coupling. This coupling, which is realized via the glutamate pool of microcolumns, triggers a cascade of downstream effects that ultimately lead to the establishment and active maintenance of the critical regime.

Thus, taking all available pieces of evidence into account, a profound new insight takes shape, namely, that *the fundamental principle behind the formation of conscious states is the resonant coupling of the brain to the ZPF*. During periods of unconsciousness, a pronounced deviation from critical dynamics is observed, implying that the coupling of the brain to the ZPF is disrupted and the ZPF, the hidden orchestrator of brain activity, is disengaged. This new insight suggests that the omnipresent ZPF is central to the understanding of consciousness.

To explore the significance of the ZPF in the formation of conscious states in more detail, let us take a look at the effects of the brain-ZPF coupling mechanism on the ZPF. In its ground state, the ubiquitous field can be described as a randomly fluctuating ocean of energy in which no field mode is singled out and no correlations exist between the field modes (De la Peña and Cetto, 1994, 1995). As discussed in Section 3, the resonant coupling of the ZPF to the glutamate pool of a microcolumn induces a phase transition that causes the dominant modes to be amplified, meaning that the amplitude of the dynamically relevant modes is considerably enhanced. Provided that the ignition criterion is satisfied, the phase transition culminates in a stationary state, entailing a frequency shift of the dominant modes to the microwave range. This shift depends on the average glutamate concentration of the ignited microcolumn, resulting in the ICMF, a microwave field with a microcolumn-specific frequency. Consequently, the occurrence of a synchronized activity pattern involving a particular set of dynamically interacting microcolumns is accompanied by a particular set of strongly correlated, amplified ZPF modes. In other words, the emergence of a synchronized activity pattern, indicative of the formation of a conscious state, entails a transition from the stochastic ZPF ground state to a modified ZPF state in which a set of modes has undergone boosting, suggesting that *the necessary condition for the formation of a conscious state is the selective amplification of ZPF modes*. For this condition to arise, a number of vital preconditions must be fulfilled along the causal chain, in particular the initiation of a phase transition, the emergence of macroscopic quantum coherence in the glutamate pool as well as the establishment and maintenance of the critical regime.

These conditions are the starting point for the development of a ZPF-based theory of consciousness, termed TRAZE in reference to the resonant amplification of the zero-point modes (Keppler, 2024). TRAZE opens up new metaphysical perspectives by linking consciousness to the foundations of physics and by creating a solid conceptual basis on which the formation of conscious states can be coherently explained. These metaphysical considerations, however, are not the subject of this work, which is explicitly confined to the neurophysiology of consciousness. We can conclude at this point that the strength of the QED-based approach lies in bringing to light a universal principle behind conscious processes and drawing a clear dividing line between conscious and unconscious brain activity, in such a way that special conditions have to be met for conscious states to occur. These conditions imply that the emergence of differentiated conscious states is tied to a unique organizing principle and that consciousness is restricted to those systems that manage to enter into a resonant interaction with the ZPF.

## 5 Empirical substantiation of the inferred operating principle

The insights gained are the result of a novel synthesis of findings from brain architecture and neurophysiology, supplemented with quantitative model calculations. The causal relationships unveiled pave the way for pioneering experimental paradigms designed to systematically manipulate conditions in the brain so as to collect new data that can be used to further empirically substantiate the inferred principle behind conscious processes.

Since the principle relies on the interaction of the brain with electromagnetic radiation in the THz range, it seems promising at first glance to apply brain stimulation methods. On closer inspection, however, this experimental path is not feasible, since THz radiation, due to its strong interaction with water molecules, penetrates only a few hundred micrometers into biological tissue and therefore cannot reach the interior of the skull (Nikitkina et al., 2021). Evolution has found a clever means of exploiting THz radiation, namely in such a way that the brain offers a suitable environment for harnessing the ZPF, i.e., the ubiquitous zero-point fluctuations of the electromagnetic field, which is why new experimental strategies directly targeting the ZPF are called for (Keppler, 2024).

**Figure 4:**
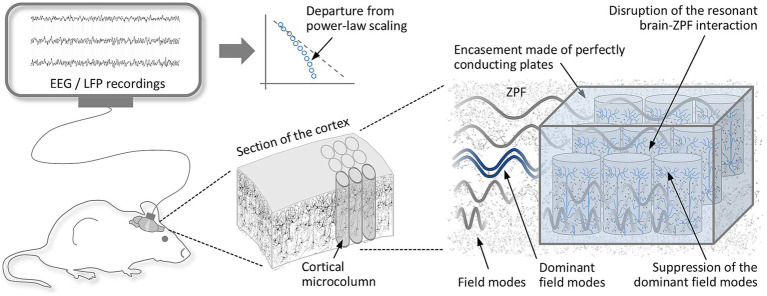
Prevention of self-organized criticality by disrupting resonant brain-ZPF coupling. By encasing arrays of microcolumns with perfectly conducting plates, the dominant field modes that are essential for the glutamate-ZPF interaction can be suppressed. Implanting a number of these encasements in a small section of the cortex is expected to prevent the initiation of phase transitions and the establishment of the critical regime in this area. This should be reflected in the analysis of activity patterns in such a way that neuronal avalanches show a clear departure from the usual power-law scaling behavior.

An immediate consequence following from the inferred principle is that SOC breaks down and conscious states cannot be formed in circumstances where the interaction of the brain with the ZPF is disrupted and there is no modification of the stochastic ZPF ground state. This results in a strategy that aims to prevent resonant brain-ZPF coupling in a selected brain area and to demonstrate that the neurophysiological characteristics of consciousness do not occur under these conditions. In other words, if it is possible to suppress, in the microcolumns of a small brain area, the dominant ZPF modes (7.8 THz) that are crucial for the glutamate-ZPF interaction, thereby blocking the initiation of phase transitions, a significant deviation from critical dynamics should be observed. This approach takes advantage of the fact that even though the ZPF can never be switched off as a whole, its frequency spectrum can be tuned locally. Such tuning can be accomplished by encasing an array of microcolumns with thin, perfectly conducting plates, a setup commonly employed in experiments on the Casimir effect (Lamoreaux, 2005). The plates impose constraints on the ZPF, so that specific frequency bands can be excluded by appropriately adjusting the plate spacing. Calculations reveal that an encasement extending over a 3-by-3 array of microcolumns has the proper dimensions to suppress the dominant ZPF modes and prevent glutamate-ZPF coupling (Keppler, 2024). Borrowing from experiments used to study neuronal avalanches (Gireesh and Plenz, 2008), an obvious approach would be to implant a number of encasements into the somatosensory cortex of rodents and to analyze the dynamical properties of activity patterns. A departure from the usual power-law scaling behavior of the avalanches would indicate that SOC, a key indicator of consciousness, depends on glutamate-ZPF coupling. Figure 4 shows a sketch of the experimental setup. In performing the experiment, the primary challenge is to avoid damaging neuronal connections when implanting the plates into the cortical tissue, which means that some technical subtleties still need to be worked out before the test can be realized (Keppler, 2024).

In addition to the neurophysiological effects, the phenomenological alterations caused by the suppression of the glutamate-ZPF interaction can be investigated in an expansion stage of the experiment. For these studies, too, the somatosensory cortex is ideally suited because it is strictly somatotopically organized, which is reflected in a precise mapping of body regions to cortical regions (Sanchez Panchuelo et al., 2018). If the body regions associated with the encased microcolumns are stimulated, the conscious states typical of these regions (e.g., the experience of pain or the feeling of touch) should not occur. The absence of conscious perceptions can be deduced from the rodents’ behavior. Once sufficient experience has been collected with the experimental setup and the test design has proven to be successful, studies on the human brain may be conducted in the future. In this case, experience reports from test subjects can be used to confirm the controlled inhibition of conscious perceptions (Keppler, 2024).

Given affirmative results, the test scenarios outlined above would lend further support to the notion that conscious states arise from the coupling of the brain to the ZPF rather than being the product of neural information processing. This would lead to a completely new understanding of consciousness and lay the groundwork for systematic research into the role of the ZPF in the formation of differentiated conscious states.

Another strategy to corroborate the ZPF-based operating principle behind conscious processes is the direct verification of quantum coherence in the glutamate pool of a microcolumn. To this end, efficient methods can be used to obtain robust indicators of quantum coherence in biological systems (Li et al., 2012), which essentially amount to measuring the populations of the vibrational states of glutamate.

Finally, there is the possibility of indirectly confirming quantum coherence by focusing on the measurement of a phenomenon that accompanies the emergence of the macroscopic quantum state of the glutamate pool. This concomitant phenomenon is the emission of photon pulses, known as biophoton emission, which arises as a result of the energy reduction of the collective state of the molecules compared to the classical state and can be detected using established experimental methods (Popp et al., 1994; Cohen and Popp, 1997; Popp, 2003). It is worth mentioning in this context that experiments have already been conducted in the brains of mice to demonstrate biophotonic activity. The results show that a significant increase in biophoton pulses is observed when highly concentrated glutamate is administered (Tang and Dai, 2014). Moreover, the experiments reveal that this biophotonic activity can be induced exclusively by glutamate, while the other neurotransmitters and neuromodulators have an enhancing or attenuating effect on the glutamate-induced activity (Chai et al., 2018). These discoveries are compatible with the ZPF-based mechanisms described above and support the theory that resonant glutamate-ZPF coupling takes place in the brain, leading to macroscopic quantum coherence in the glutamate pool.

## 6 Discussion and outlook

In summary, we have drawn on the methods of quantum field theory to explore brain dynamics associated with conscious states. In its basics, this approach reaches back to theoretical studies dealing with the emergence of long-range correlations and macroscopic order, initially with a focus on memory and the identification of memory states with macroscopic ordered states created and maintained by quantum interactions (Ricciardi and Umezawa, 1967; Stuart et al., 1979). In physical terms, stable macroscopic organizational patterns correspond to states of minimal energy that result from spontaneous symmetry breaking. Based on these originally very general ideas and considerations, subsequent works delved into the mechanisms behind symmetry breaking, indicating that the formation of macroscopic ordered states in a system can be attributed to the interaction of the system components with the ubiquitous vacuum fluctuations of the electromagnetic field, here referred to as ZPF, and that the electromagnetic field plays a crucial stabilizing role in maintaining ordered states (Preparata, 1995; Del Giudice et al., 2005; Del Giudice and Vitiello, 2006). Beyond the application to memory, the identified mechanisms have also been discussed in the context of consciousness (Vitiello, 1995, 2001), in particular with regard to the explanation of the neural activity patterns related to conscious perception, which originate from symmetry-breaking phase transitions and bear the hallmarks of SOC (Freeman and Vitiello, 2006, 2008). In these studies, however, it remained open which components in the brain are decisive for the coupling to the ZPF, how phase transitions are initiated, and what exact regulatory mechanisms lie behind SOC. The QED-based model calculations summarized in Sections 3 and 4 help to shed light on these details and provide deeper insights into the organizational principles involved in macroscopic ordering phenomena in the brain. From this perspective, the present work can be seen as a systematic advancement of quantum field-theoretical approaches to understanding brain dynamics.

The methodology pursued here is to be distinguished from so-called quantum-like models, which have their origin in quantum information theory and aim to describe the behavior of complex systems using the mathematical formalism of quantum theory (Khrennikov, 2023). This approach is based on the hypothesis that complex systems, such as the brain, are information processors whose processing characteristics obey the laws of quantum information theory. Using this formalism, which has been applied to psychological and cognitive problems, particularly in the area of decision making (Khrennikov, 2009; Asano et al., 2012; Busemeyer et al., 2014), the system behavior is modeled at a meta-level at which the detailed physical processes are disregarded. However, a physical model for the functioning of the brain has been proposed within this meta-level framework that reproduces the laws of quantum information theory and could be the basis for quantum-like processing (Khrennikov, 2011). The crucial element of this classical model is a random electromagnetic background field produced by a huge number of neurons. This points to conceptual similarities between the description of cognitive meta-level processes and the description of the fundamental physical processes underlying the formation of the neural activity patterns associated with conscious states, in which the ZPF, i.e., the presence of random vacuum fluctuations of the electromagnetic field, is the decisive element.

Returning to the main findings, there is growing evidence that SOC, the key neurophysiological characteristic of conscious processes, is based on the brain’s interaction with the ZPF, with the ZPF being instrumental in the homeostatic regulation of the neuronal firing rate and the fine-tuning of the E-I balance. In contrast, the signature of unconscious brain activity, such as that induced by anesthetics, is a significant deviation from critical dynamics (Alonso et al., 2014; Tagliazucchi et al., 2016; Toker et al., 2022; Maschke et al., 2024). In the following, we will take a closer look at the mode of action of anesthetics.

Anesthetics can exert their effect via various pathways, including ligand-gated ion channels, voltage-gated ion channels, and also microtubules (MTs), which are important elements of the cytoskeleton (Kelz and Mashour, 2019). Much research has focused on ligand-gated ion channels as the presumed primary targets of anesthetic action (Franks and Lieb, 1994). Studies show that volatile anesthetics elevate GABA_A_ receptor-mediated inhibitory synaptic transmission and depress NMDA receptor-mediated excitatory synaptic transmission (Wakasugi et al., 1999). The inhibitory path has been explored extensively, demonstrating that anesthetics bind to the transmembrane domain of the GABA_A_ receptor, resulting in conformational changes of transmembrane proteins and inducing channel activation (Li et al., 2006; Chen et al., 2018; Woll et al., 2018). Model calculations also reveal that anesthetic binding to channel proteins alters the gating motion and influences the conductance of the ion channels (Bertaccini, 2010).

In the light of these findings, the mode of action of anesthetics can be explained by the fact that they modify GABA_A_ receptor-mediated and NMDA receptor-mediated synaptic transmission and thus disturb the fine-tuned E-I balance, which has detrimental effects on the establishment and maintenance of synchronized activity patterns (Faulkner et al., 1998) and results in deviations from critical behavior (Fekete et al., 2018). From the perspective of the fundamental principle behind conscious processes discussed in Section 4, it can be concluded that under the altered conditions the coordinating function of the ZPF is impaired, as the field cannot properly fulfill its central role in regulating the neuronal firing rate and orchestrating neuronal avalanches.

Furthermore, experiments suggest that, in addition to their influence on ion channels, certain types of general anesthetics target MTs and that the destabilization of MTs is a contributary factor to anesthesia (Emerson et al., 2013). This is supported by a recent study, indicating that the binding of the anesthetic gas isoflurane to MTs contributes to unconsciousness in rats (Khan et al., 2024). MTs play a crucial part in the maintenance of synaptic functions as they control the replenishment of synaptic modules. More specifically, MTs serve as the main structures along which synaptic components, such as synaptic vesicle precursors (SVPs), are transported (Aiken and Holzbaur, 2021). This entails that a disruption of MT dynamics leads to a markedly reduced supply of SVPs to the synapses (Miryala et al., 2022), which explains that the anesthetic-induced impairment of the functioning of MTs affects glutamate release in the synapses, preventing the initiation of phase transitions and the establishment of the critical regime.

As far as the specific mechanism of action of anesthetics is concerned, it has long been hypothesized that macroscopic quantum coherence in MTs may be crucial for the formation of conscious states (Hameroff, 1994; Hameroff et al., 2014). This hypothesis forms the backbone of the Orch OR theory, according to which conscious events are associated with orchestrated quantum processes in neuronal MTs (Hameroff and Penrose, 2014), a path that has recently been taken up again (Wiest, 2025). It is assumed that the binding of anesthetic gases to tubulin, the protein subunit of MTs, inhibits the formation of collective modes (quantum dipole vibrations) and thus disrupts macroscopic quantum coherence, which is supported by experimental findings indicating that the *π*-electron clouds of the aromatic amino acids of tubulin display characteristic collective oscillations and that anesthetics induce shifts of the characteristic frequencies (Craddock et al., 2017).

The importance of MTs for sustaining consciousness dovetails with the notion that synchronized neural activity patterns, which are indicative of conscious states, result from a bottom-up orchestration process driven by the ZPF. As previously described, this process involves the glutamate-ZPF coupling in ignited microcolumns, the amplification of the dominant ZPF modes culminating in the ICMF, the ICMF-based regulation of the neuronal firing rate, and the controlled propagation of synchronized action potentials. It seems plausible that this process continues downward to the MTs, implying that the dynamical properties of the MTs, which are responsible for the proper transport of glutamate to the synaptic vesicles, are governed by the coupling of MT components to the ZPF. To underpin this idea, it is necessary to take a closer look at the MT-ZPF interaction, to study the dynamical features of the coupled MT-ZPF system using model calculations, and to investigate the possibility of functionally relevant ZPF-mediated macroscopic quantum effects in MTs.

Beyond that, a more detailed physical model of voltage-gated ion channels is yet to be developed, allowing us to describe how microwaves modulate the ion flow through these channels, regulate the firing rate of neurons, and achieve the E-I balance. Equipped with such a model, further experimental strategies can be derived to test the postulated ICMF-based mechanism behind SOC.

Finally, it should be pointed out that the QED-based approach discussed here not only lays a solid theoretical foundation for explaining brain dynamics but also sheds new light on the regulation of biological organisms as a whole. The principles set forth in this article therefore give reason to conjecture that the resonant interaction between specific molecules and the ZPF, resulting in the formation of coherence domains and the generation of endogenous electromagnetic fields, could be the basis of many homeostatic control processes taking place in cells. From this vantage point, each control process utilizes unique frequencies or frequency bands, and a healthy organism can be thought of as a perfectly coordinated orchestra.

## Data Availability

The original contributions presented in the study are included in the article/supplementary material, further inquiries can be directed to the corresponding author.

## References

[ref1] Aiken J. Holzbaur E. L. F. (2021). Cytoskeletal regulation guides neuronal trafficking to effectively supply the synapse. Curr. Biol. 31, R633–R650. doi: 10.1016/j.cub.2021.02.024, PMID: 34033795 PMC8360495

[ref2] Alonso L. M. Proekt A. Schwartz T. H. Pryor K. O. Cecchi G. A. Magnasco M. O. (2014). Dynamical criticality during induction of anesthesia in human ECoG recordings. Front. Neural Circuits 8:20. doi: 10.3389/fncir.2014.00020, PMID: 24723852 PMC3971201

[ref3] Arani R. Bono I. Del Giudice E. Preparata G. (1995). QED coherence and the thermodynamics of water. Int. J. Mod. Phys. B 9, 1813–1841. doi: 10.1142/S0217979295000744

[ref4] Asano M. Basieva I. Khrennikov A. Ohya M. Tanaka Y. (2012). Quantum-like dynamics of decision-making. Phys. A 391, 2083–2099. doi: 10.1016/j.physa.2011.11.042

[ref5] Bartos M. Vida I. Jonas P. (2007). Synaptic mechanisms of synchronized gamma oscillations in inhibitory interneuron networks. Nat. Rev. Neurosci. 8, 45–56. doi: 10.1038/nrn2044, PMID: 17180162

[ref6] Beggs J. M. Plenz D. (2003). Neuronal avalanches in neocortical circuits. J. Neurosci. 23, 11167–11177. doi: 10.1523/JNEUROSCI.23-35-11167.2003, PMID: 14657176 PMC6741045

[ref7] Bertaccini E. J. (2010). The molecular mechanisms of anesthetic action: updates and cutting edge developments from the field of molecular modeling. Pharmaceuticals 3, 2178–2196. doi: 10.3390/ph3072178, PMID: 27713348 PMC4036663

[ref8] Bono I. Del Giudice E. Gamberale L. Henry M. (2012). Emergence of the coherent structure of liquid water. Water 4, 510–532. doi: 10.3390/w4030510

[ref9] Bouganne R. Bosch Aguilera M. Ghermaoui A. Beugnon J. Gerbier F. (2020). Anomalous decay of coherence in a dissipative many-body system. Nat. Phys. 16, 21–25. doi: 10.1038/s41567-019-0678-2

[ref10] Busemeyer J. R. Wang Z. Khrennikov A. Basieva I. (2014). Applying quantum principles to psychology. Phys Scr T163:014007. doi: 10.1088/0031-8949/2014/T163/014007

[ref11] Buxhoeveden D. P. Casanova M. F. (2002). The minicolumn hypothesis in neuroscience. Brain 125, 935–951. doi: 10.1093/brain/awf110, PMID: 11960884

[ref12] Buzsáki G. Wang X. J. (2012). Mechanisms of gamma oscillations. Annu. Rev. Neurosci. 35, 203–225. doi: 10.1146/annurev-neuro-062111-150444, PMID: 22443509 PMC4049541

[ref13] Cetto A. M. de la Peña L. (2022). The electromagnetic vacuum field as an essential hidden ingredient of the quantum-mechanical ontology. Entropy 24:1717. doi: 10.3390/e24121717, PMID: 36554122 PMC9777925

[ref14] Chai W. Han Z. Wang Z. Li Z. Xiao F. Sun Y. . (2018). Biophotonic activity and transmission mediated by mutual actions of neurotransmitters are involved in the origin and altered states of consciousness. Neurosci. Bull. 34, 534–538. doi: 10.1007/s12264-018-0215-9, PMID: 29508252 PMC5960451

[ref15] Chen Q. Wells M. M. Arjunan P. Tillman T. S. Cohen A. E. Xu Y. . (2018). Structural basis of neurosteroid anesthetic action on GABA_A_ receptors. Nat. Commun. 9:3972. doi: 10.1038/s41467-018-06361-4, PMID: 30266951 PMC6162318

[ref16] Chialvo D. R. (2010). Emergent complex neural dynamics. Nat. Phys. 6, 744–750. doi: 10.1038/nphys1803

[ref17] Cohen S. Popp F. A. (1997). Biophoton emission of the human body. J. Photochem. Photobiol. B Biol. 40, 187–189. doi: 10.1016/s1011-1344(97)00050-x, PMID: 9345786

[ref18] Craddock T. J. A. Kurian P. Preto J. Sahu K. Hameroff S. R. Klobukowski M. . (2017). Anesthetic alterations of collective terahertz oscillations in tubulin correlate with clinical potency: implications for anesthetic action and post-operative cognitive dysfunction. Sci. Rep. 7:9877. doi: 10.1038/s41598-017-09992-7, PMID: 28852014 PMC5575257

[ref19] De la Peña L. Cetto A. M. (1994). Quantum phenomena and the zeropoint radiation field. Found. Phys. 24, 917–948. doi: 10.1007/BF02067655

[ref20] De la Peña L. Cetto A. M. (1995). Quantum phenomena and the zeropoint radiation field II. Found. Phys. 25, 573–604. doi: 10.1007/BF02059007

[ref21] De la Peña L. Cetto A. M. Valdés-Hernández A. (2015). The emerging quantum. The physics behind quantum mechanics. Cham: Springer International Publishing.

[ref22] Del Giudice E. De Ninno A. Fleischmann M. Mengoli G. Milani M. Talpo G. . (2005). Coherent quantum electrodynamics in living matter. Electromagn. Biol. Med. 24, 199–210. doi: 10.1080/15368370500379574

[ref23] Del Giudice E. Doglia S. Milani M. Vitiello G. (1985). A quantum field theoretical approach to the collective behaviour of biological systems. Nucl. Phys. B. 251, 375–400. doi: 10.1016/0550-3213(85)90267-6

[ref24] Del Giudice E. Tedeschi A. Vitiello G. Voeikov V. (2013). Coherent structures in liquid water close to hydrophilic surfaces. J. Phys. Conf. Ser. 442:012028. doi: 10.1088/1742-6596/442/1/012028

[ref25] Del Giudice E. Vitiello G. (2006). Role of the electromagnetic field in the formation of domains in the process of symmetry-breaking phase transitions. Phys. Rev. A 74:022105. doi: 10.1103/PhysRevA.74.022105

[ref26] Doesburg S. M. Green J. J. McDonald J. J. Ward L. M. (2009). Rhythms of consciousness: binocular rivalry reveals large-scale oscillatory network dynamics mediating visual perception. PLoS One 4:e6142. doi: 10.1371/journal.pone.0006142, PMID: 19582165 PMC2702101

[ref27] Emerson D. J. Weiser B. P. Psonis J. Liao Z. Taratula O. Fiamengo A. . (2013). Direct modulation of microtubule stability contributes to anthracene general anesthesia. J. Am. Chem. Soc. 135, 5389–5398. doi: 10.1021/ja311171u, PMID: 23484901 PMC3671381

[ref28] Engel A. K. Singer W. (2001). Temporal binding and the neural correlates of sensory awareness. Trends Cogn. Sci. 5, 16–25. doi: 10.1016/S1364-6613(00)01568-0, PMID: 11164732

[ref29] Erecińska M. Silver I. A. (1990). Metabolism and role of glutamate in mammalian brain. Prog. Neurobiol. 35, 245–296. doi: 10.1016/0301-0082(90)90013-7, PMID: 1980745

[ref30] Faulkner H. J. Traub R. D. Whittington M. A. (1998). Disruption of synchronous gamma oscillations in the rat hippocampal slice: a common mechanism of anaesthetic drug action. Br. J. Pharmacol. 125, 483–492. doi: 10.1038/sj.bjp.0702113, PMID: 9806331 PMC1565655

[ref31] Featherstone D. E. (2010). Intercellular glutamate signaling in the nervous system and beyond. ACS Chem. Neurosci. 1, 4–12. doi: 10.1021/cn900006n, PMID: 22778802 PMC3368625

[ref32] Fekete T. Omer D. B. O’Hashi K. Grinvald A. van Leeuwen C. Shriki O. (2018). Critical dynamics, anesthesia and information integration: lessons from multi-scale criticality analysis of voltage imaging data. NeuroImage 183, 919–933. doi: 10.1016/j.neuroimage.2018.08.026 30120988

[ref33] Fingelkurts A. A. Fingelkurts A. A. Neves C. F. H. (2013). Consciousness as a phenomenon in the operational architectonics of brain organization: criticality and self-organization considerations. Chaos Solitons Fractals 55, 13–31. doi: 10.1016/j.chaos.2013.02.007

[ref34] Franks N. P. Lieb W. R. (1994). Molecular and cellular mechanisms of general anaesthesia. Nature 367, 607–614. doi: 10.1038/367607a0, PMID: 7509043

[ref35] Freeman W. J. (2004). Origin, structure, and role of background EEG activity. Part 2. Analytic phase. Clin. Neurophysiol. 115, 2089–2107. doi: 10.1016/j.clinph.2004.02.028, PMID: 15294211

[ref36] Freeman W. J. (2007). Indirect biological measures of consciousness from field studies of brains as dynamical systems. Neural Netw. 20, 1021–1031. doi: 10.1016/j.neunet.2007.09.004, PMID: 17923391

[ref37] Freeman W. J. Vitiello G. (2006). Nonlinear brain dynamics as macroscopic manifestation of underlying many-body field dynamics. Phys Life Rev 3, 93–118. doi: 10.1016/j.plrev.2006.02.001

[ref38] Freeman W. J. Vitiello G. (2008). “The dissipative quantum model of brain and laboratory observations” in Physics of emergence and organization. eds. Licata I. Sakaji A. (Singapore: World Scientific), 233–251.

[ref39] Fries P. Nikolić D. Singer W. (2007). The gamma cycle. Trends Neurosci. 30, 309–316. doi: 10.1016/j.tins.2007.05.005, PMID: 17555828

[ref40] Gaillard R. Dehaene S. Adam C. Clemenceau S. Hasboun D. Baulac M. . (2009). Converging intracranial markers of conscious access. PLoS Biol. 7:e1000061. doi: 10.1371/journal.pbio.1000061, PMID: 19296722 PMC2656551

[ref41] Gireesh E. D. Plenz D. (2008). Neuronal avalanches organize as nested theta and beta/gamma-oscillations during development of cortical layer 2/3. Proc. Natl. Acad. Sci. USA 105, 7576–7581. doi: 10.1073/pnas.0800537105, PMID: 18499802 PMC2396689

[ref42] Hameroff S. R. (1994). Quantum coherence in microtubules: a neural basis for emergent consciousness. J. Conscious. Stud. 1, 91–118.

[ref43] Hameroff S. R. Craddock T. J. A. Tuszynski J. A. (2014). Quantum effects in the understanding of consciousness. J. Integr. Neurosci. 13, 229–252. doi: 10.1142/S0219635214400093, PMID: 25012711

[ref44] Hameroff S. Penrose R. (2014). Consciousness in the universe: a review of the ‘Orch OR’ theory. Phys Life Rev 11, 39–78. doi: 10.1016/j.plrev.2013.08.002, PMID: 24070914

[ref45] Hosoya T. (2019). The basic repeating modules of the cerebral cortical circuit. Proc. Jpn. Acad. Ser. B Phys. Biol. Sci. 95, 303–311. doi: 10.2183/pjab.95.022, PMID: 31406055 PMC6766449

[ref46] Jones E. G. (2000). Microcolumns in the cerebral cortex. Proc. Natl. Acad. Sci. USA 97, 5019–5021. doi: 10.1073/pnas.97.10.5019, PMID: 10805761 PMC33979

[ref47] Kelso J. Bressler S. Buchanan S. DeGuzman G. Ding M. Fuchs A. . (1992). A phase transition in human brain and behavior. Phys. Lett. A 169, 134–144. doi: 10.1016/0375-9601(92)90583-8

[ref48] Kelz M. B. Mashour G. A. (2019). The biology of general anesthesia from paramecium to primate. Curr. Biol. 29, R1199–R1210. doi: 10.1016/j.cub.2019.09.071, PMID: 31743680 PMC6902878

[ref49] Keppler J. (2016). On the universal mechanism underlying conscious systems and the foundations for a theory of consciousness. Open J. Phil. 6, 346–367. doi: 10.4236/ojpp.2016.64034

[ref50] Keppler J. (2018). The role of the brain in conscious processes: a new way of looking at the neural correlates of consciousness. Front. Psychol. 9:1346. doi: 10.3389/fpsyg.2018.01346, PMID: 30123156 PMC6085561

[ref51] Keppler J. (2021). Building blocks for the development of a self-consistent electromagnetic field theory of consciousness. Front. Hum. Neurosci. 15:723415. doi: 10.3389/fnhum.2021.723415, PMID: 34650416 PMC8505726

[ref52] Keppler J. (2023). Scrutinizing the feasibility of macroscopic quantum coherence in the brain: a field-theoretical model of cortical dynamics. Front. Phys. 11:1181416. doi: 10.3389/fphy.2023.1181416

[ref53] Keppler J. (2024). Laying the foundations for a theory of consciousness: the significance of critical brain dynamics for the formation of conscious states. Front. Hum. Neurosci. 18:1379191. doi: 10.3389/fnhum.2024.1379191, PMID: 38736531 PMC11082359

[ref54] Keppler J. (2025). The fundamental principle underlying conscious processes and necessary conditions for the formation of conscious states. ResearchGate (preprint). doi: 10.13140/RG.2.2.26179.72480

[ref55] Keppler J. Shani I. (2020). Cosmopsychism and consciousness research: a fresh view on the causal mechanisms underlying phenomenal states. Front. Psychol. 11:371. doi: 10.3389/fpsyg.2020.00371, PMID: 32210886 PMC7066492

[ref56] Khan S. Huang Y. Timuçin D. Bailey S. Lee S. Lopes J. . (2024). Microtubule-stabilizer epothilone B delays anesthetic-induced unconsciousness in rats. ENeuro 11:ENEURO.0291-24.2024. doi: 10.1523/ENEURO.0291-24.2024, PMID: 39147581 PMC11363512

[ref57] Khrennikov A. (2009). Quantum-like model of cognitive decision making and information processing. Biosystems 95, 179–187. doi: 10.1016/j.biosystems.2008.10.004, PMID: 19010383

[ref58] Khrennikov A. (2011). Quantum-like model of processing of information in the brain based on classical electromagnetic field. Biosystems 105, 250–262. doi: 10.1016/j.biosystems.2011.05.014, PMID: 21683119

[ref59] Khrennikov A. (2023). Open quantum Systems in Biology, cognitive and social sciences. Cham: Springer.

[ref60] Kitzbichler M. G. Smith M. L. Christensen S. R. Bullmore E. (2009). Broadband criticality of human brain network synchronization. PLoS Comput. Biol. 5:e1000314. doi: 10.1371/journal.pcbi.1000314, PMID: 19300473 PMC2647739

[ref61] Knyazev G. G. Slobodskoj-Plusnin J. Y. Bocharov A. V. Pylkova L. V. (2011). The default mode network and EEG alpha oscillations: an independent component analysis. Brain Res. 1402, 67–79. doi: 10.1016/j.brainres.2011.05.052, PMID: 21683942

[ref62] Koch C. Hepp K. (2006). Quantum mechanics in the brain. Nature 440, 611–612. doi: 10.1038/440611a, PMID: 16572152

[ref63] Kohn A. Pinheiro A. Tommerdahl M. A. Whitsel B. L. (1997). Optical imaging *in vitro* provides evidence for the minicolumnar nature of cortical response. Neuroreport 8, 3513–3517. doi: 10.1097/00001756-199711100-00019, PMID: 9427317

[ref64] Lamoreaux S. K. (2005). The Casimir force: background, experiments, and applications. Rep. Prog. Phys. 68, 201–236. doi: 10.1088/0034-4885/68/1/R04

[ref65] Li G. D. Chiara D. C. Sawyer G. W. Husain S. S. Olsen R. W. Cohen J. B. (2006). Identification of a GABA_A_ receptor anesthetic binding site at subunit interfaces by photolabeling with an etomidate analog. J. Neurosci. 26, 11599–11605. doi: 10.1523/JNEUROSCI.3467-06.2006, PMID: 17093081 PMC6674783

[ref66] Li C. Lambert N. Chen Y. Chen G. Nori F. (2012). Witnessing quantum coherence: from solid-state to biological systems. Sci. Rep. 2:885. doi: 10.1038/srep00885, PMID: 23185690 PMC3505870

[ref67] Li X. Liu C. Liang W. Ye H. Chen W. Lin R. . (2014). Millimeter wave promotes the synthesis of extracellular matrix and the proliferation of chondrocyte by regulating the voltage-gated K+ channel. J. Bone Miner. Metab. 32, 367–377. doi: 10.1007/s00774-013-0513-2, PMID: 24202060

[ref68] Linkenkaer-Hansen K. Nikouline V. V. Palva J. M. Ilmoniemi R. J. (2001). Long-range temporal correlations and scaling behavior in human brain oscillations. J. Neurosci. 21, 1370–1377. doi: 10.1523/JNEUROSCI.21-04-01370.2001, PMID: 11160408 PMC6762238

[ref69] Lombardi F. Herrmann H. J. Plenz D. De Arcangelis L. (2014). On the temporal organization of neuronal avalanches. Front. Syst. Neurosci. 8:204. doi: 10.3389/fnsys.2014.00204, PMID: 25389393 PMC4211381

[ref70] Ma Z. Turrigiano G. G. Wessel R. Hengen K. B. (2019). Cortical circuit dynamics are homeostatically tuned to criticality *in vivo*. Neuron 104, 655–664.e4. doi: 10.1016/j.neuron.2019.08.031, PMID: 31601510 PMC6934140

[ref71] Marder E. Goaillard J. M. (2006). Variability, compensation and homeostasis in neuron and network function. Nat. Rev. Neurosci. 7, 563–574. doi: 10.1038/nrn1949, PMID: 16791145

[ref72] Maschke C. O’Byrne J. Colombo M. A. Boly M. Gosseries O. Laureys S. . (2024). Critical dynamics in spontaneous EEG predict anesthetic-induced loss of consciousness and perturbational complexity. Commun. Biol. 7:946. doi: 10.1038/s42003-024-06613-8, PMID: 39103539 PMC11300875

[ref73] Mazzoni A. Broccard F. D. Garcia-Perez E. Bonifazi P. Ruaro M. E. Torre V. (2007). On the dynamics of the spontaneous activity in neuronal networks. PLoS One 2:e439. doi: 10.1371/journal.pone.0000439, PMID: 17502919 PMC1857824

[ref74] Melloni L. Molina C. Pena M. Torres D. Singer W. Rodriguez E. (2007). Synchronization of neural activity across cortical areas correlates with conscious perception. J. Neurosci. 27, 2858–2865. doi: 10.1523/JNEUROSCI.4623-06.2007, PMID: 17360907 PMC6672558

[ref75] Mewes C. Pellegrin S. Fleischhauer M. Kurizki G. (2005). “Coherence protection near energy gaps” in Decoherence, entanglement and information protection in complex quantum systems. eds. Akulin V. Sarfati A. Kurizki G. Pellegrin S. (Dordrecht: Springer Netherlands), 201–222.

[ref76] Milicevic K. D. Barbeau B. L. Lovic D. D. Patel A. A. Ivanova V. O. Antic S. D. (2023). Physiological features of parvalbumin-expressing GABAergic interneurons contributing to high-frequency oscillations in the cerebral cortex. Curr. Res. Neurobiol. 6:100121. doi: 10.1016/j.crneur.2023.100121 38616956 PMC11015061

[ref77] Miryala C. S. J. Holland E. D. Dent E. W. (2022). Contributions of microtubule dynamics and transport to presynaptic and postsynaptic functions. Mol. Cell. Neurosci. 123:103787. doi: 10.1016/j.mcn.2022.103787, PMID: 36252720 PMC9838116

[ref78] Modolo J. Hassan M. Wendling F. Benquet P. (2020). Decoding the circuitry of consciousness: from local microcircuits to brain-scale networks. Netw. Neurosci. 4, 315–337. doi: 10.1162/netn_a_00119, PMID: 32537530 PMC7286300

[ref79] Mountcastle V. B. (1997). The columnar organization of the neocortex. Brain 120, 701–722. doi: 10.1093/brain/120.4.701, PMID: 9153131

[ref80] Nikitkina A. I. Bikmulina P. Y. Gafarova E. R. Kosheleva N. V. Efremov Y. M. Bezrukov E. A. . (2021). Terahertz radiation and the skin: a review. J. Biomed. Opt. 26:043005. doi: 10.1117/1.JBO.26.4.043005, PMID: 33583155 PMC7881098

[ref81] Páscoa dos Santos F. Verschure P. F. M. J. (2025). Excitatory-inhibitory homeostasis and bifurcation control in the Wilson-Cowan model of cortical dynamics. PLoS Comput. Biol. 21:e1012723. doi: 10.1371/journal.pcbi.1012723, PMID: 39761317 PMC11737862

[ref82] Pickard W. F. Rosenbaum F. J. (1978). Biological effects of microwaves at the membrane level: two possible athermal electrophysiological mechanisms and a proposed experimental test. Math. Biosci. 39, 235–253. doi: 10.1016/0025-5564(78)90055-X

[ref83] Pikov V. Arakaki X. Harrington M. Fraser S. E. Siegel P. H. (2010). Modulation of neuronal activity and plasma membrane properties with low-power millimeter waves in organotypic cortical slices. J. Neural Eng. 7:045003. doi: 10.1088/1741-2560/7/4/045003, PMID: 20644247

[ref84] Pinotsis D. A. Fridman G. Miller E. K. (2023). Cytoelectric coupling: electric fields sculpt neural activity and “tune” the brain’s infrastructure. Prog. Neurobiol. 226:102465. doi: 10.1016/j.pneurobio.2023.102465, PMID: 37210066 PMC12302380

[ref85] Plenz D. Ribeiro T. L. Miller S. R. Kells P. A. Vakili A. Capek E. L. (2021). Self-organized criticality in the brain. Front. Phys. 9:639389. doi: 10.3389/fphy.2021.639389

[ref86] Plenz D. Thiagarajan T. C. (2007). The organizing principles of neuronal avalanches: cell assemblies in the cortex? Trends Neurosci. 30, 101–110. doi: 10.1016/j.tins.2007.01.005, PMID: 17275102

[ref87] Popp F. A. (2003). “Biophotons — background, experimental results, theoretical approach and applications” in Integrative biophysics. eds. Popp F. A. Beloussov L. (Dordrecht: Springer), 387–438.

[ref88] Popp F. A. Gu Q. Li K. H. (1994). Biophoton emission: experimental background and theoretical approaches. Mod. Phys. Lett. B 8, 1269–1296. doi: 10.1142/s0217984994001266

[ref89] Preparata G. (1995). QED coherence in matter. Singapore: World Scientific Publishing.

[ref90] Rey A. M. Jiang L. Fleischhauer M. Demler E. Lukin M. D. (2008). Many-body protected entanglement generation in interacting spin systems. Phys. Rev. A 77:052305. doi: 10.1103/PhysRevA.77.052305

[ref91] Ricciardi L. M. Umezawa H. (1967). Brain and physics of many-body problems. Kybernetik 4, 44–48. doi: 10.1007/BF00292170, PMID: 5617419

[ref92] Rodriguez E. George N. Lachaux J. P. Martinerie J. Renault B. Varela F. J. (1999). Perception’s shadow: long distance synchronization of human brain activity. Nature 397, 430–433. doi: 10.1038/17120, PMID: 9989408

[ref93] Salkoff D. B. Zagha E. Yuzgec O. McCormick D. A. (2015). Synaptic mechanisms of tight spike synchrony at gamma frequency in cerebral cortex. J. Neurosci. 35, 10236–10251. doi: 10.1523/JNEUROSCI.0828-15.2015, PMID: 26180200 PMC4502264

[ref94] Sanchez Panchuelo R. M. Besle J. Schluppeck D. Humberstone M. Francis S. (2018). Somatotopy in the human somatosensory system. Front. Hum. Neurosci. 12:235. doi: 10.3389/fnhum.2018.00235, PMID: 29950980 PMC6008546

[ref95] Schmidt-Wilcke T. Fuchs E. Funke K. Vlachos A. Müller-Dahlhaus F. Puts N. A. J. . (2018). GABA—from inhibition to cognition: emerging concepts. Neuroscientist 24, 501–515. doi: 10.1177/1073858417734530, PMID: 29283020

[ref96] Shani I. Keppler J. (2018). Beyond combination: how cosmic consciousness grounds ordinary experience. J. Am. Philos. Assoc. 4, 390–410. doi: 10.1017/apa.2018.30

[ref97] Shneider M. N. Pekker M. (2014). Initiation and blocking of the action potential in an axon in weak ultrasonic or microwave fields. Phys. Rev. E 89:052713. doi: 10.1103/PhysRevE.89.052713, PMID: 25353835

[ref98] Spruston N. (2008). Pyramidal neurons: dendritic structure and synaptic integration. Nat. Rev. Neurosci. 9, 206–221. doi: 10.1038/nrn2286, PMID: 18270515

[ref99] Steinmann S. Leicht G. Ertl M. Andreou C. Polomac N. Westerhausen R. . (2014). Conscious auditory perception related to long-range synchrony of gamma oscillations. NeuroImage 100, 435–443. doi: 10.1016/j.neuroimage.2014.06.012, PMID: 24945670

[ref100] Stewart C. V. Plenz D. (2006). Inverted-u profile of dopamine-NMDA-mediated spontaneous avalanche recurrence in superficial layers of rat prefrontal cortex. J. Neurosci. 26, 8148–8159. doi: 10.1523/JNEUROSCI.0723-06.2006, PMID: 16885228 PMC6673780

[ref101] Stuart C. I. J. M. Takahashi Y. Umezawa H. (1979). Mixed system brain dynamics: neural memory as a macroscopic ordered state. Found. Phys. 9, 301–327. doi: 10.1007/BF00715185

[ref102] Tagliazucchi E. Balenzuela P. Fraiman D. Chialvo D. R. (2012). Criticality in large-scale brain fMRI dynamics unveiled by a novel point process analysis. Front. Physiol. 3:15. doi: 10.3389/fphys.2012.00015, PMID: 22347863 PMC3274757

[ref103] Tagliazucchi E. Chialvo D. R. Siniatchkin M. Amico E. Brichant J. F. Bonhomme V. . (2016). Large-scale signatures of unconsciousness are consistent with a departure from critical dynamics. J. R. Soc. Interface 13:20151027. doi: 10.1098/rsif.2015.1027, PMID: 26819336 PMC4759808

[ref104] Tang R. Dai J. (2014). Spatiotemporal imaging of glutamate-induced biophotonic activities and transmission in neural circuits. PLoS One 9:e85643. doi: 10.1371/journal.pone.0085643, PMID: 24454909 PMC3893221

[ref105] Tegmark M. (2000). Importance of quantum decoherence in brain processes. Phys. Rev. E 61, 4194–4206. doi: 10.1103/PhysRevE.61.4194, PMID: 11088215

[ref106] Toker D. Pappas I. Lendner J. D. Frohlich J. Mateos D. M. Muthukumaraswamy S. . (2022). Consciousness is supported by near-critical slow cortical electrodynamics. Proc. Natl. Acad. Sci. USA 119:e2024455119. doi: 10.1073/pnas.2024455119, PMID: 35145021 PMC8851554

[ref107] Uhlhaas P. J. Pipa G. Lima B. Melloni L. Neuenschwander S. Nikolić D. . (2009). Neural synchrony in cortical networks: history, concept and current status. Front. Integr. Neurosci. 3:17. doi: 10.3389/neuro.07.017.2009, PMID: 19668703 PMC2723047

[ref108] Vitiello G. (1995). Dissipation and memory capacity in the quantum brain model. Int. J. Mod. Phys. B 9, 973–989. doi: 10.1142/S0217979295000380

[ref109] Vitiello G. (2001). My double unveiled. Amsterdam: John Benjamins.

[ref110] Wakasugi M. Hirota K. Roth S. H. Ito Y. (1999). The effects of general anesthetics on excitatory and inhibitory synaptic transmission in area CA1 of the rat hippocampus in vitro. Anesth. Analg. 88, 676–680. doi: 10.1097/00000539-199903000-00039, PMID: 10072027

[ref111] Walter N. Hinterberger T. (2022). Self-organized criticality as a framework for consciousness: a review study. Front. Psychol. 13:911620. doi: 10.3389/fpsyg.2022.911620, PMID: 35911009 PMC9336647

[ref112] Wen W. Turrigiano G. G. (2024). Keeping your brain in balance: homeostatic regulation of network function. Annu. Rev. Neurosci. 47, 41–61. doi: 10.1146/annurev-neuro-092523-110001, PMID: 38382543

[ref113] Wiest M. C. (2025). A quantum microtubule substrate of consciousness is experimentally supported and solves the binding and epiphenomenalism problems. Neurosci. Conscious. 2025:niaf011. doi: 10.1093/nc/niaf011, PMID: 40342554 PMC12060853

[ref114] Woll K. A. Zhou X. Bhanu N. V. Garcia B. A. Covarrubias M. Miller K. W. . (2018). Identification of binding sites contributing to volatile anesthetic effects on GABA type a receptors. FASEB J. 32, 4172–4189. doi: 10.1096/fj.201701347R, PMID: 29505303 PMC6044061

[ref115] Wu Y. K. Hengen K. B. Turrigiano G. G. Gjorgjieva J. (2020). Homeostatic mechanisms regulate distinct aspects of cortical circuit dynamics. Proc. Natl. Acad. Sci. USA 117, 24514–24525. doi: 10.1073/pnas.1918368117, PMID: 32917810 PMC7533694

